# “At Least the Second Plate”: rethinking nutritional care in the geriatric unit

**DOI:** 10.1016/j.jnha.2026.100887

**Published:** 2026-06-02

**Authors:** Emanuele Marzetti, Riccardo Calvani, Hélio Jose Coelho-Junior

**Affiliations:** a A. Gemelli" University Hospital Foundation (IRCCS), Rome, Italy; b Department of Geriatrics, Orthopedics and Rheumatology, Catholic University of the Sacred Heart, Rome, Italy

**Keywords:** Sarcopenia, Malnutrition, Frailty, Protein intake, Caloric intake

## Abstract

Adequate nutritional care is important to reduce adverse outcomes in hospitalized older adults. However, achieving adequate nutritional intake in geriatric units is challenging due to appetite-related disorders, chronic diseases, anabolic resistance, and limitations associated with oral nutritional supplementation. In this context, healthcare professionals often encourage patients to consume “at least the second plate,” which is usually centered on protein-rich foods, as an attempt to preserve muscle mass and support recovery. However, it remains unclear whether this strategy alone is sufficient to meet the nutritional needs of hospitalized older adults. In this manuscript, we discuss the limitations of focusing predominantly on protein-rich meals and emphasize the importance of adequate energy intake for muscle preservation and overall recovery in geriatric inpatients.

Adequate nutritional support is crucial strategy for reducing the risk of adverse outcomes among patients in the geriatric unit [[1], [2], [3]]. However, nutritional interventions in hospitalized older adults are often challenging due to the coexistence of appetite-related disorders, including anorexia of aging, dysgeusia, dysphagia, xerostomia, and the specific nutritional requirements associated with chronic conditions (e.g., diabetes, hypertension, chronic kidney disease) [[4], [5], [6]]. The acknowledgement of muscle loss as an important clinical entity owing to its involvement in sarcopenia, frailty and malnutrition provides further complications to patient management, as the ingestion of high-quality protein is necessary to support muscle anabolism [7,8].

Results from pooled analysis and cohort studies indicate that benefits are reached when protein ingestion exceeds the current Recommended Dietary Allowance (RDA) (0.8 g/kg of body weight [BW]) [[9], [10], [11], [12], [13], [14]]. These findings encouraged international organizations responsible for people’s care and nutrition to recommend a daily protein intake of 1.0 to 1.2 g/kg of BW/day for healthy older adults, while higher amounts 1.2–1.5 g/kg of BW/day are endorsed for those with acute or chronic illnesses, malnutrition, and/or severe injury [7,8,15]. Such intake patterns are difficult to achieve exclusively with protein from main meals, leading to the adoption of oral nutrition as a complementary approach. Nevertheless, barriers associated with the utilization of oral supplements (e.g., gastrointestinal problems, negative connotation, costs, medical team management) still hamper the adequate intake of proteins in this population [16,17].

As a consequence, the consumption of the whole meal during hospitalization becomes a priority. Again, the combination of appetite-related disorders and food characteristics (e.g., flavor, texture, aroma) [18] limits optimal food intake. In these circumstances, nutritionists and dietitians frequently recommend patients and caregivers to consume “at least the second plate”, which is traditionally centered on a protein-rich dish. A question that emerges is whether this approach is sufficient to support muscle mass maintenance and overall recovery.

While adequate protein intake is essential to support muscle homeostasis and stimulate muscle protein synthesis, it does not represent the sole nutritional consideration for improving patient outcomes. Energy restriction commonly promotes muscle loss, as a calorie deficit can disrupt muscle protein balance, favoring protein breakdown. This occurs because amino acids are diverted for gluconeogenesis and used as fuel by various tissues [19]. From a theoretical perspective, increasing protein intake during conditions of calorie deficit may help counteract muscle catabolism and attenuate the loss of lean mass [19]. However, empirical evidence suggests that the effectiveness of this approach is contingent upon the relationship between calorie deficit severity and amount of dietary protein.

For instance, Pasiakos et al. [20] compared the effects of three dietary protein interventions (0.8, 1.6, and 2.4 g/kg of BW/day) in adults undergoing 21-day 40% energy deficit. Authors observed that only protein intake threefold higher than the RDA (i.e., 2.4 g/kg of BW/day) significantly increased postprandial muscle protein synthesis. Though, this increase was insufficient to preserve fat-free mass, as significant reductions were observed across all study groups, particularly at the lowest protein intake levels (58.2%, 29.8%, and 36.4%, respectively). Similar findings were reported by Hector et al. [21] after examining the effects of low (1.2 g/kg of BW/day) and high (2.4 g/kg of BW/day) protein intake diets during 10 days of 40%-reduced energy intake. Results indicated significant losses in lean mass, regardless of protein ingestion levels. More recently, Oxfeldt et al. [22] showed that low energy availability – a concept that combines calorie restriction and increased energy expenditure – reduces myofibrillar and sarcoplasmic protein synthesis and decreases nitrogen balance, thereby promoting losses of fat- and bone-free lean mass.

Despite the specific characteristics of each service, in general, daily hospital meals provide nearly 2,000 kcal with approximately 79 g of protein [23]. Nevertheless, patients in acute care settings fail to consume nearly one quarter of the calories (524 kcal) and protein (24 g) offered, resulting in 84.4% of patients not achieving recommended nutritional intake targets [23]. This level of protein intake, approximately 55 g/day, may be sufficient for very low-weight older adults and non-malnourished individuals weighing up to 55 kg – providing 1.0 g of protein per kg of BW/day – but is likely inadequate for heavier or malnourished patients.

Importantly, this estimate accounts for protein intake across all meals, indicating that the nutritional deficit may be even greater when considering only the second plate. Indeed, patients on bed rest commonly experience a reduction in insulin sensitivity [24], which contributes to the development of anabolic resistance — a condition in which amino acid uptake is impaired [25,26], indicating that higher protein intake may be necessary to stimulate muscle anabolism.

The scenario is even more concerning when considering the nutritional composition of second plates. For instance, a serving of grilled chicken breast (80–120 g), commonly provided in Western hospitals, offers approximately 165 kcal when fully consumed. If consumed at both lunch and dinner, these meals would provide nearly 330 kcal, indicating that eutrophic patients would still need to consume an additional 1,000–2,000 kcal through the remaining meals to meet their daily energy requirements, according to ESPEN recommendations [15,27].

Furthermore, prioritizing protein intake without adequate consideration of total energy and macronutrient balance may overlook the central role of calorie intake in whole-body functioning and the impact of energy deficit and hypoglycemia on geriatric syndromes, including cognitive decline, fatigue, and delirium, potentially complicating clinical diagnosis and management [28,29].

Taken together, this evidence indicates that energy intake is a key determinant of muscle homeostasis, as energy deficit enhances muscle proteolysis by suppressing muscle protein synthesis and driving a negative protein balance. Although prioritizing the second plate may increase protein provision, evidence suggests that this alone may still be insufficient when total energy intake is low. Indeed, the literature shows that under conditions of substantial energy deficit (40%), even protein intakes up to three times the current RDA may fail to preserve muscle mass, and may also contribute to additional clinical complications.

Hence, individualized nutritional interventions are required to ensure adequate energy and macronutrient intake, thereby reducing the risk of complications. Although broad recommendations are challenging, two main approaches emerge. First, nutritionists and dietitians should encourage the combination of first and second plates, even in patients with reduced appetite. Relying solely on the second plate may increase protein intake but is unlikely to fully meet protein requirements. In such cases, protein deficits may be corrected with oral nutritional supplements when necessary. Second, although supervised progressive resistance training protocols are still scarcely adopted in geriatric units despite strong supporting evidence [30], muscle contraction is a key stimulus for muscle protein synthesis [19], and studies suggest that exercise training may help maintain or even increase muscle mass even in the context of very-low-calorie diets [21].

In conclusion, the available evidence indicates that adequate nutritional care in hospitalized older adults requires a broader approach than protein provision alone. While protein intake remains essential for muscle maintenance, energy availability is an equally critical determinant of muscle homeostasis and overall physiological function. Focusing exclusively on the “second plate” may therefore be insufficient, as it overlooks the importance of total energy intake and its impact on clinical outcomes. Nutritional strategies should combine adequate energy provision with sufficient high-quality protein intake, tailored to individual patient needs, to prevent or attenuate muscle loss and related complications. A combined approach integrating dietary optimization with exercise training may offer the most effective strategy to preserve muscle mass and improve recovery in geriatric inpatients.

## CRediT authorship contribution statement

All authors contributed to all aspects of the manuscript preparation, including conceiving the review topic, performing the literature search, and drafting the manuscript. All authors also contributed to editing, critically reviewed the manuscript for important intellectual content, and approved the final version.

## Ethical standards

This article is based exclusively on previously published literature and does not include any new studies involving human participants or animals conducted by the authors. Therefore, ethical approval and informed consent were not required. Clinical trial registration: not applicable. Consent to participate: not applicable. Consent for publication: not applicable.

## Declaration of Generative AI and AI-assisted technologies in the writing process

No artificial intelligence (AI) tools were utilized during the preparation of this manuscript.

## Funding

Not applicable.

## Data availability statement

All data utilized in this manuscript are available in the cited refereces.

## Declaration of competing interest

All data utilized in this manuscript are available in the cited references.
